# Using Chromatin Accessibility to Delineate Therapeutic Subtypes in Pancreatic Cancer Patient-Derived Cell Lines

**DOI:** 10.1016/j.xpro.2020.100079

**Published:** 2020-08-04

**Authors:** Holly Brunton, Ian M. Garner, Ulla-Maja Bailey, Rosie Upstill-Goddard, Peter J. Bailey

**Affiliations:** 1 Institute of Cancer Sciences, University of Glasgow, Garscube Estate, Switchback Road, Bearsden, Glasgow G61 1QH, UK; 2 Cancer Research UK Beatson Institute, Garscube Estate, Switchback Road, Glasgow G61 1BD, UK; 3 Epigenetics Unit, Department of Surgery & Cancer, Imperial College London, Hammersmith Campus, Du Cane Road, London W12 0NN, UK; 4 Department of General surgery, University of Heidelberg, 69120 Heidelberg, Germany

## Abstract

Disrupted chromatin regulatory processes contribute to the development of cancer, in particular pancreatic ductal adenocarcinoma. The assay for transposase accessible chromatin with high-throughput sequencing (ATAC-seq) is typically used to study chromatin organization. Here, we present a revised ATAC-seq protocol to study chromatin accessibility in adherent patient-derived cell lines. We provide details on how to calculate the library molarity using Agilent’s Bioanalyzer and an analysis pipeline for peak calling and transcription factor mapping.

For complete details on the use and execution of this protocol, please refer to Brunton et al. (2020).

## Highlights

• ATAC-seq protocol to measure chromatin accessibility in patient-derived cell lines
• Includes detailed size selection library clean-up step
• Instructions on how to calculate the library molarity using Agilent Bioanalyzer
• A comprehensive data analysis pipeline is provided

## Graphical Abstract

**Figure:**
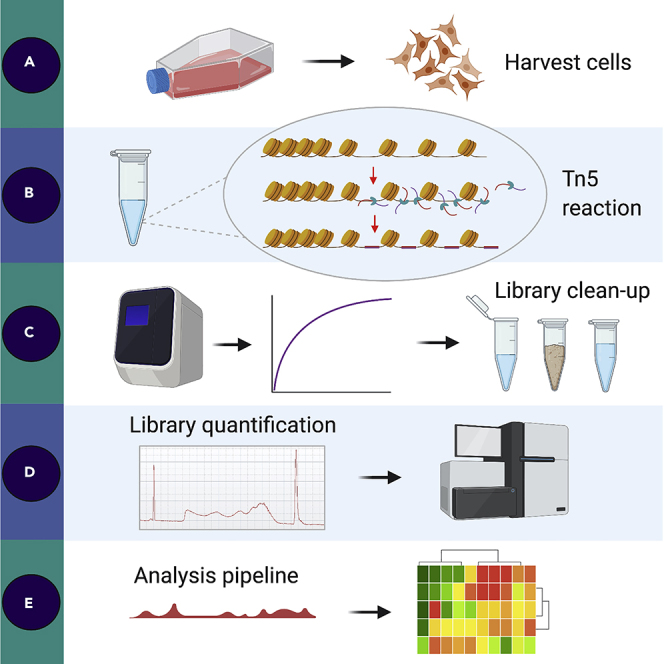

## Before You Begin

***Note:*** This protocol has been modified from Buenrostro et al. (2015).

Additional benefits incorporated in this revised protocol include:

1. A double size selection library clean-up step.
2. Detailed library quantification using Agilent’s Bioanalyzer.
3. A complete ATAC-seq data analysis pipeline for peak calling and TF mapping.

This protocol has been specifically optimized for adherent pancreatic cancer patient-derived cell lines (PDCLs), but we have also successfully used it in cell lines derived from Genetically Modified Mouse Models (GEMMs), such as the LSL-Kras^G12D/+^;LSL-Trp53^R172H/+^;Pdx-1-Cre (KPC) mouse model. Refer to the Troubleshooting section for guidelines on optimizations for different cell types.

1. Cell lines should be grown for at least 48 h undisrupted before start of assay. Ensure cells have adapted to media and any dead cells are removed.
2. Resuspend all adapter sequences to 25 μM working stock concentration. See Table 1 for sequences.

3. Prepare fresh ATAC-seq lysis buffer (10 mM Tris-HCl, pH 7.4, 10 mM NaCl, 3 mM MgCl_2_, 0.1% IGEPAL).
4. On day of assay, pre-chill centrifuge to 4°C and set thermal mixer to 37°C.

**Table 1:** ATAC-Seq Oligos Used for PCR

Oligo Name	Oligo Sequence (5′ to 3′)
Ad1_noMX	AATGATACGGCGACCACCGAGATCTACACTCGTC GGCAGCGTCAGATGTG
Ad2.1	CAAGCAGAAGACGGCATACGAGATTCGCCTTAGT CTCGTGGGCTCGGAGATGT
Ad2.2	CAAGCAGAAGACGGCATACGAGATCTAGTACGGT CTCGTGGGCTCGGAGATGT
Ad2.3	CAAGCAGAAGACGGCATACGAGATTTCTGCCTGT CTCGTGGGCTCGGAGATGT
Ad2.4	CAAGCAGAAGACGGCATACGAGATGCTCAGGAGT CTCGTGGGCTCGGAGATGT
Ad2.5	CAAGCAGAAGACGGCATACGAGATAGGAGTCCGT CTCGTGGGCTCGGAGATGT
Ad2.6	CAAGCAGAAGACGGCATACGAGATCATGCCTAGT CTCGTGGGCTCGGAGATGT
Ad2.7	CAAGCAGAAGACGGCATACGAGATGTAGAGAGGT CTCGTGGGCTCGGAGATGT
Ad2.8	CAAGCAGAAGACGGCATACGAGATCCTCTCTGGT CTCGTGGGCTCGGAGATGT
Ad2.9	CAAGCAGAAGACGGCATACGAGATAGCGTAGCGT CTCGTGGGCTCGGAGATGT
Ad2.10	CAAGCAGAAGACGGCATACGAGATCAGCCTCGGT CTCGTGGGCTCGGAGATGT
Ad2.11	CAAGCAGAAGACGGCATACGAGATTGCCTCTTGT CTCGTGGGCTCGGAGATGT
Ad2.12	CAAGCAGAAGACGGCATACGAGATTCCTCTACGT CTCGTGGGCTCGGAGATGT
Ad2.13	CAAGCAGAAGACGGCATACGAGATATCACGACGT CTCGTGGGCTCGGAGATGT
Ad2.14	CAAGCAGAAGACGGCATACGAGATACAGTGGTGT CTCGTGGGCTCGGAGATGT
Ad2.15	CAAGCAGAAGACGGCATACGAGATCAGATCCAGT CTCGTGGGCTCGGAGATGT
Ad2.16	CAAGCAGAAGACGGCATACGAGATACAAACGGGT CTCGTGGGCTCGGAGATGT
Ad2.17	CAAGCAGAAGACGGCATACGAGATACCCAGCAGT CTCGTGGGCTCGGAGATGT
Ad2.18	CAAGCAGAAGACGGCATACGAGATAACCCCTCGT CTCGTGGGCTCGGAGATGT
Ad2.19	CAAGCAGAAGACGGCATACGAGATCCCAACCTGT CTCGTGGGCTCGGAGATGT
Ad2.20	CAAGCAGAAGACGGCATACGAGATCACCACACGT CTCGTGGGCTCGGAGATGT
Ad2.21	CAAGCAGAAGACGGCATACGAGATGAAACCCAGT CTCGTGGGCTCGGAGATGT
Ad2.22	CAAGCAGAAGACGGCATACGAGATTGTGACCAGT CTCGTGGGCTCGGAGATGT
Ad2.23	CAAGCAGAAGACGGCATACGAGATAGGGTCAAGT CTCGTGGGCTCGGAGATGT
Ad2.24	CAAGCAGAAGACGGCATACGAGATAGGAGTGGGT CTCGTGGGCTCGGAGATGT

Custom made primers were purchased from ThermoFisher Scientific using 200 nmol synthesis scale (200N) and desalted (DSL) purification.

## Key Resources Table

**Table:** 

REAGENT or RESOURCE	SOURCE	IDENTIFIER
**Chemicals, Peptides, and Recombinant Proteins**		

SYBR Select master Mix	ThermoFisher	Cat# 4472903

**Critical Commercial Assays**		

Illumina Tagment DNA TDE1 Enzyme and Buffer kit	Illumina	Cat# 20034197
MinElute PCR Purification Kit	Qiagen	Cat# 28004
NEBNext High-Fidelity 2× PCR Master Mix	New England Biolabs	Cat# M0541S
Agencourt AMPure XP beads	Fisher Scientific	Cat# 10136224
Bioanalyzer DNA analysis	Agilent Technologies	Cat# 5067
AffinityScript Multiple temperature cDNA synthesis kit	Agilent Technologies	Cat# 200436

**Software and Algorithms**		

HOMER	(Heinz et al., 2010)	http://homer.ucsd.edu/homer/
STRING	(Szklarczyk et al., 2015)	http://string-db.org
ChipSeeker	(Yu et al., 2015)	https://bioconductor.org/packages/release/bioc/html/ChIPseeker.html
Bcbio-nextgen toolkit	GitHub	https://bcbio-nextgen.readthedocs.io/en/latest/
DiffBind	(Ross-Innes et al., 2012)	https://bioconductor.org/packages/release/bioc/html/DiffBind.html
Clipper	Bioconductor	https://bioconductor.org/packages/release/bioc/html/clipper.html

## Step-by-Step Method Details

### Cell Preparation

**Timing: 10–20 min**

Cells are trypsinized, counted, and lysed with ATAC-seq lysis buffer.

***Note:*** This protocol is a modification of a previous protocol by Buenrostro et al. (2015).

***Note:*** Optimizing the cell number to transposase enzyme ratio is crucial to obtain DNA libraries of sufficient complexity. For example, over-transposition of chromatin results when too few cells are used and produces a library composed of mainly small DNA fragments (<500 bp). Under-transposition results when too many cells are used and generates mainly large difficult to sequence DNA fragments (>1,000 bp).

1. Harvest PDCLs using trypsin and count cells
a. Typically, 100,000 cells worked well for most PDCLs tested. To optimize this for different cell types a range of cell numbers should be tested first to establish optimal cell: transposase ratio.
b. Pipette cells using a 200 μL pipette tip to produce homogenous single cell suspension.
2. Centrifuge 100,000 cells for 5 min at 600 × *g*, 4°C.
3. Remove and discard supernatant.
4. Add 50 μL ice-cold PBS to wash pellet, do not disturb pellet, and centrifuge for 5 min at 600 × *g*, 4°C.
5. Remove and discard supernatant.
6. Add 50 μL ice-cold ATAC-seq lysis buffer to pellet. Very gently dislodge the pellet. Centrifuge immediately for 10 min at 600 × *g*, 4°C.

**CRITICAL:** It is crucial to treat the cell pellet gently during the lysis step to prevent over-lysis and preserve the native chromatin configuration to be analyzed.

**CRITICAL:** To proceed as quickly as possible to the transposition reaction, make the transposition master mix (described in step 9) while the pellet is spinning for 10 min at 600 × *g*, 4°C (step 6)

7. Discard the supernatant and immediately continue to the transposition reaction.

### Transposition Reaction and Purification

**Timing: ∼45 min**

Cells are incubated with the Tn5 transposase enzyme to insert oligonucleotide tags within open regions of DNA.

8. Make sure the cell pellet is placed on ice.
9. To make the transposition reaction mix, combine the following:
25 μL TD (2× reaction buffer from Nextera kit)
4.7 μL TDE1 (Nextera Tn5 Transposase from Nextera kit)
22.3 μL nuclease-free H_2_O
10. Resuspend nuclei pellet in the transposition reaction mix.
11. Incubate the transposition reaction at 37°C for 30 min at 400 rpm on an Eppendorf mixer.
12. Immediately following transposition, purify the DNA using the Qiagen MiniElute PCR purification kit.
13. Elute transposed DNA in 10 μL DNase-free water.

**Pause Point:** Purified DNA can be stored at −20°C for up to 72 h.

### PCR Amplification

**Timing: 30–60 min**

Transposed DNA fragments are amplified by PCR using custom primers which contain the designated index (barcode) for each library.

***Note:*** Each library must contain a unique index to enable sample identification once the libraries have been pooled for sequencing.

14. To amplify transposed DNA fragments, combine the following in a 0.2 mL PCR tube:
10 μL transposed DNA
10 μL nuclease-free H_2_O
2.5 μL 25 μM PCR Primer 1
2.5 μL 25 μM Barcoded PCR primer 2
25 μL NEBNext High-Fidelity 2× PCR master mix
15. Perform initial amplification as follows:

**Table:** 

Step	Temperature	Time
1	72°C	0:05
2	98°C	0:30
3	98°C	0:10
4	63°C	0:30
5	72°C	1:00
6	Go to step 3, 5× more times	
7	4°C	forever

**CRITICAL:** To allow extension of both ends of the primer after transposition the first 5 min extension at 72°C is required.

***Note:*** The final number of PCR cycles is determined using qPCR to allow library amplification to be stopped prior to saturation. This helps to reduce GC and size biases.

16. To run a qPCR side reaction, combine the following qPCR compatible consumables:
5 μL of previously PCR-amplified DNA
4.2 μL H_2_O
0.4 μL 25 μM primer 1
0.4 μL 25 μM primer 2
5 μL 2× SYBR Select master Mix

**CRITICAL:** Use white qPCR plates to run the qPCR reaction. White plates help increase sensitivity and reduce variability in the qPCR assay.

17. Using a qPCR instrument, cycle as follows:

**Table:** 

Step	Temperature	Time
1	98°C	0:30
2	98°C	0:10
	63°C	0:30
	72°C	1:00
3	Go to step 2, 20× more times

**Figure 1:**
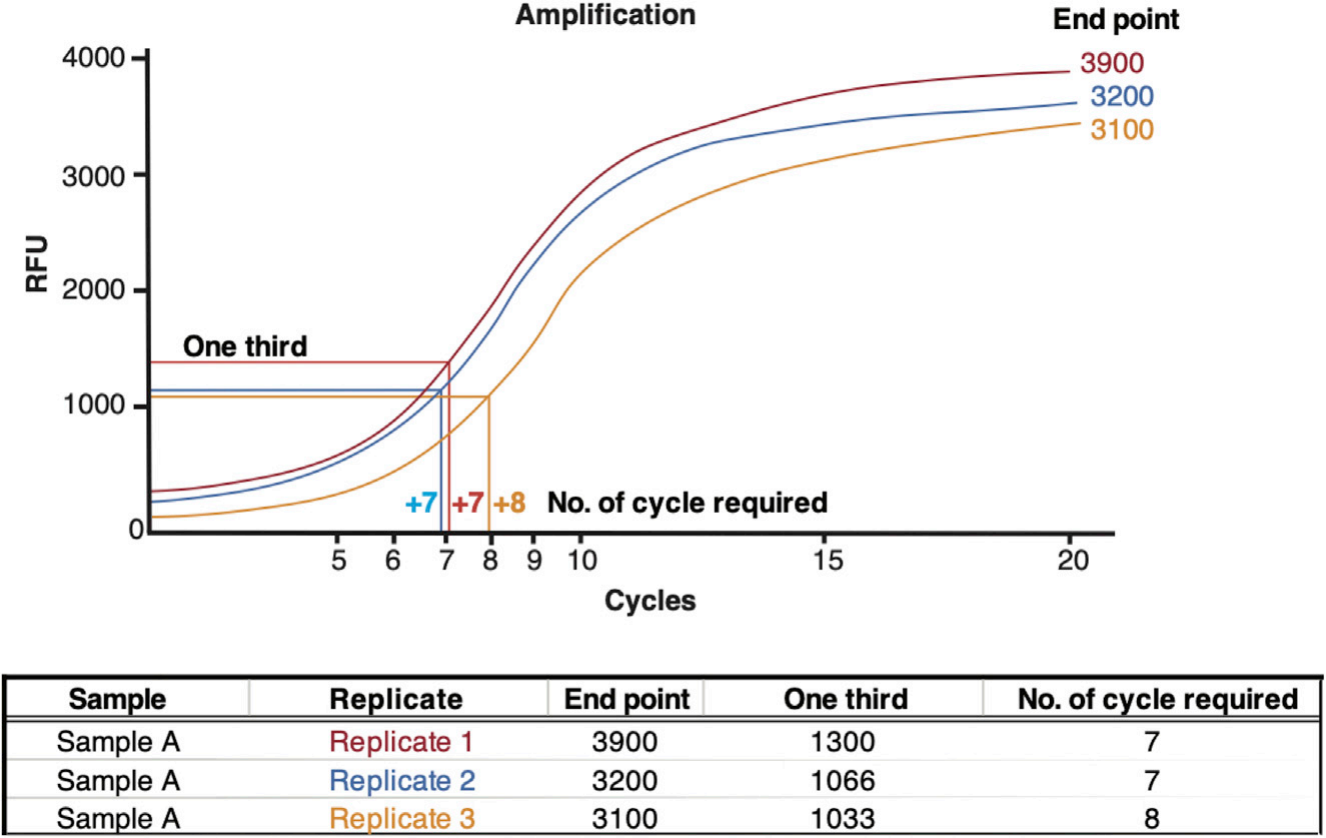
Calculating the Number of Additional PCR Cycles Required for Library Amplification To calculate the additional number of cycles required, plot linear Rn versus cycle and determine the additional number that corresponds to one-third of the maximum fluorescent intensity. For example, as shown for sample A replicate 1, the “end point” RFU value after 20 cycles is 3,900 which is divided by 3 to give “one third” RFU value of 1,300. A line is then drawn at 1,300 on the y-axis until it meets the amplification curve of that sample. This point is then joined to the x-axis to establish the number of additional cycles required for the library amplification. Take forward replicate libraries which require the minimum number of cycles for library amplification.

18. To calculate the additional number of cycles required, plot linear Rn versus cycle and determine the additional number that corresponds to one-third of the maximin florescent intensity (Figure 1).

19. Run the remaining 45 μL PCR reaction to the cycle number determined by qPCR.

**Table:** 

Steps	Temperature	Time
1	98°C	0:30
2	98°C	0:10
	63°C	0:30
	72°C	1:00
3	Go to step 2, N more times

20. Purify the amplified library using Qiagen Minielute PCR purification kit.
21. Elute in 20 μL EB buffer.

### Double Size Selection

**Timing: 30–60 min**

The first objective is to remove large DNA fragments which are >1,000 bp. Large fragments are difficult to accurately quantify and result in reduced clustering efficiencies when sequencing. The second objective is to remove smaller DNA fragments which are <100 bp. Smaller fragments are preferentially sequenced and consequently could become over-represented in the sequencing result. This step will also remove excess adapter sequenced.

***Note:*** This protocol is adapted from the SPRI Based Size Selection manual

**CRITICAL:** Vortex magnetic beads and bring beads and DNA to room temperature before starting size selection assay.

**Table:** 

Reagent
80% ethanol, non-denatured (prepared fresh)
DNase-free water
Magnetic beads such as AMPure XP or SPRIselect for library clean-up and size selection
DynaMag2 magnetic stand

***Note:*** Steps 22–27 are for Large Size Selection (>1,000 bp)

22. Vortex beads and bring beads and DNA to RT.
23. Make all samples up to 50 μL using resuspension buffer (DNase-free water).
24. To select for fragments <1,000 bp use 0.55× SPRIselect ratio.

For example: 50 μL sample × 0.55× ratio = 27.5 μL of SPRIselect to add to library.

25. Add beads to sample and pipette 10 times and incubate for 5 min at RT.

**CRITICAL:** Insufficient mixing of samples and SPRIselect will lead to inconsistent size selection results.

***Note:*** The yields of ATAC-seq libraries are typically lower as compared to other library protocols, e.g., RNA-seq or WGS. To ensure that all of the desired DNA fragments are bound to the beads the incubation time can be extended. However, extending the time of DNA and bead incubation can increase binding of unwanted size fragments.

26. Add samples to an appropriate magnetic rack or plate such as DynaMag-2 Magnet. Wait for the beads to attached to the magnet and for the sample to go clear. Bead attachment times can vary depending on the initial sample size, the volume of beads added, and the strength of the magnet.
27. Take the supernatant and keep - the large unwanted fragments are attached to the beads.

**CRITICAL:** Be careful not to pipette any of the beads when removing the supernatant as this will contaminate the library prep with large DNA fragments.

***Note:*** Steps 28–32 are for Small Size Selection (>100 bp)

***Note:*** Increasing the ratio of SPRIselect volume to sample volume will decrease the efficiency of binding larger fragments.

28. To select for DNA fragments that are 100 bp and above use a final volume of 1.8× SPRIselect based on the initial reaction volume. The second SPRIselect volume is determined after accounting for the first volume of 0.55× SPRIselect added.

For example: 50 μL sample × (1.8× ratio − 0.55× ratio) = 62.5 μL SPRIselect.

29. Add SPRIselect to sample, mix well, and incubate for 5 min at RT.
30. Add sample to magnetic rack.
31. Make fresh 80% ethanol.
32. Once beads have attached to the magnet and the sample has gone clear, remove, and discard the supernatant. The library is now attached to the beads.

**CRITICAL:** Be careful not to remove beads during this step because the desired library is now associated with the beads. Significant bead loss will result in reduced yield.

33. With the reaction vessel still on the magnet, add 500 μL of 80% ethanol and incubate at RT for 1 min. Remove and discard the ethanol supernatant.

**CRITICAL:** Take care not to remove any beads during this step.

34. Carefully remove residual ethanol with a pipette and dry for 5 min at RT.

**CRITICAL:** Allow all ethanol to evaporate but DO NOT let the beads dry out.

The bead supernatant will begin to crack if allowed to dry out completely – avoid this.

35. Remove sample from magnetic rack and resuspend beads in 23 μL nuclease-free H_2_O. Mix well.

***Note:*** Elution volume should be large enough so that the liquid level is high enough for the beads to settle to the magnet.

36. Leave samples to stand off the magnetic rack for 1 min.
37. Add samples back to the magnetic rack and allow beads to attach to the magnet.
38. Once clear, take 20 μL of eluted DNA and store in a DNA-low binding Eppendorf at 4°C.

### Estimate of Library Yield Using the Qubit dsDNA HS Assay Kits

**Timing: 15 min**

To accurately quantify the ATAC-seq library we recommend using Agilent Bioanalyzer high sensitivity DNA chips. These chips are extremely sensitive and work best when 1 ng/μL library is loaded. To establish the ATAC-seq library concentration for loading onto the bioanalyzer chip, first use the Qubit dsDNA HS Assay Kits to obtain a rough estimate of library yield.

The Qubit dsDNA HS (High sensitivity) Assay Kits include concentrated assay reagent, dilution buffer and prediluted DNA standards. The assay is accurate for sample concentrations from 10 pg/μL to 100 ng/μL. This assay is prepared at room temperature (RT) and is stable for 3 h.

***Note:*** See life technologies cat# Q32851, Q32854 for Qubit dsDNA HS Assay Kits user manual.

39. Set up the required number of 0.5 mL tubes for standards and samples. The Qubit dsDNA HS assay requires 2 standards.

**CRITICAL:** Use only thin-wall, clear 0.5 mL PCR tubes. Acceptable tubes include Qubit assay tubes (cat #Q32856) or Axygen PCR-05-C tubes (cat # 10011-830).

40. Label the tube lid.

**CRITICAL:** Do not label the side of the tube as this could interfere with the sample read.

41. Prepare Qubit working solution by diluting the dsDNA HS reagent 1:200 in dsDNA HS buffer.

***Note:*** The final volume in each tube must be 200 μL. Each standard tube contains 190 μL of Qubit working solution, and each sample tube requires somewhere between 180 μL to 199 μL (depending on the concentration or your prepared library).

42. For the standards, add 10 μL standard to 190 μL Qubit working solution.
43. For samples, add 2 μL sample to 198 μL Qubit working solution.

***Note:*** Typically, 2 μL of the library prep provides a sufficient volume to give an accurate Qubit reading which fell in between the range of detection, i.e., between the two standards. However, test samples can be anywhere from 1–20 μL. Add corresponding volume of Qubit working solution to each tube: anywhere from 180–199 μL.

44. Mix all samples by vortexing then spin briefly.
45. Incubate at RT for 2 min
46. Read samples on Qubit 3.0 Fluorometer by pressing the DNA option, then select dsDNA High Sensitivity as the assay type.
47. Press Read Standards to proceed
48. Insert tube containing Standard #1. Read standard and once complete, read standard #2.
49. Press Run samples.
50. On the assay screen select sample volume and units:
Sample volume from 1–20 μL
Select the units for the output sample concentration, i.e., ng/μL
51. Read all samples.

### Library Quantification and Molarity Calculation Using the Bioanalyzer

**Timing: 2 h**

The Bioanalyzer high sensitivity DNA kits can quantify libraries from just a few amplification cycles thereby reducing the amplification bias of shorter DNA fragments and improving the quality of sequencing data. The kit also offers improved sensitivity for checking the size and quantity of DNA samples of limited abundance.

***Note:*** See protocol brochure for detailed step-by-step procedure: Manual Part Number: G2938-09321 Rev.B

**CRITICAL: Exactly 1μL of sample must be loaded. A mximum of 11 samples can be run per chip.**

The chip can detect DNA fragments in the range of 50–7,000 bp. This sizing range is optimal for analyzing the full spectrum of DNA fragments generated in ATAC-seq libraries.

Quantitative range is 5–500 pg/μL

Make sure gel matrix reagents are at RT for at least 30 min before adding to chip.

Typically, 500 pg/μL loaded onto the chip gives an accurate quantification.

**Figure 2:**
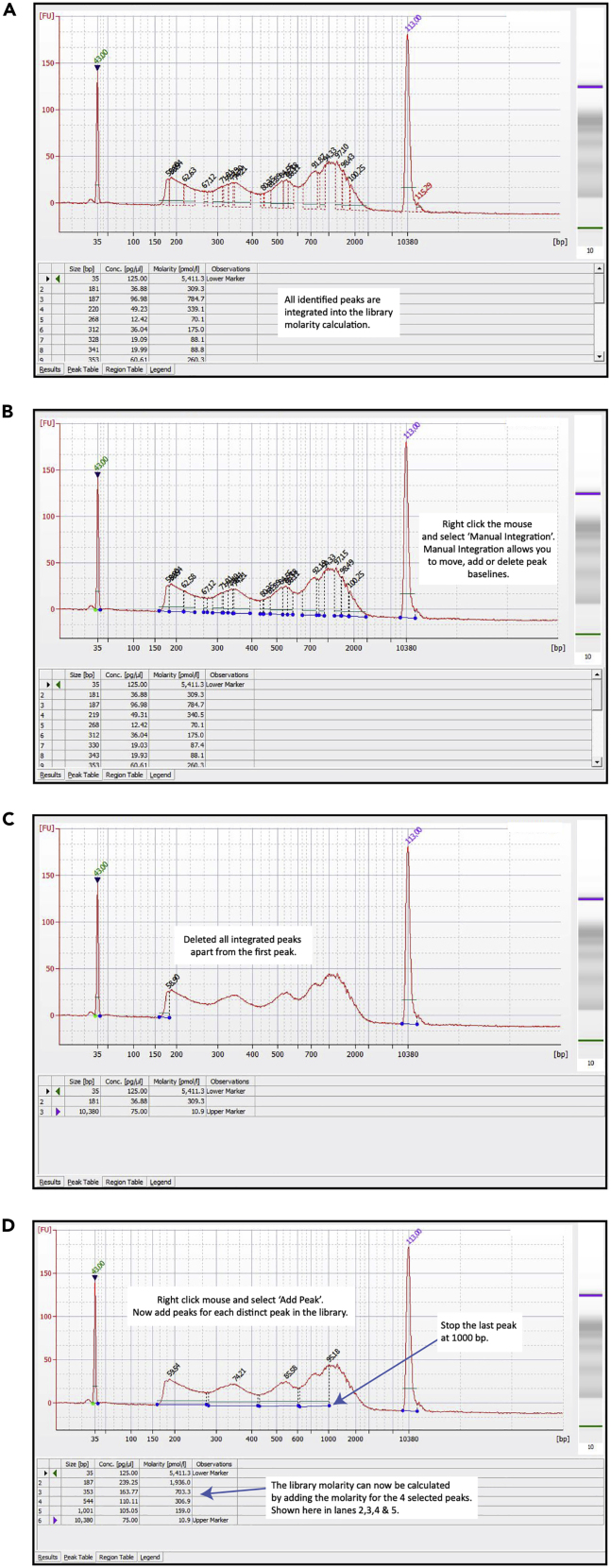
Calculating the ATA-Seq Library Molarity Using the Agilent Bioanalyzer (A) Run 0.5 ng/μL of ATAC-seq library on the Bioanalyzer. (B) Select “Manual Integration” to enable removal of integrated peaks. (C) Delete integrated peaks. (D) Now “Add Peaks” to incorporate all distinct peaks in the library. Add the molarity values for the selected peaks to establish the library molarity.

**Figure 3:**
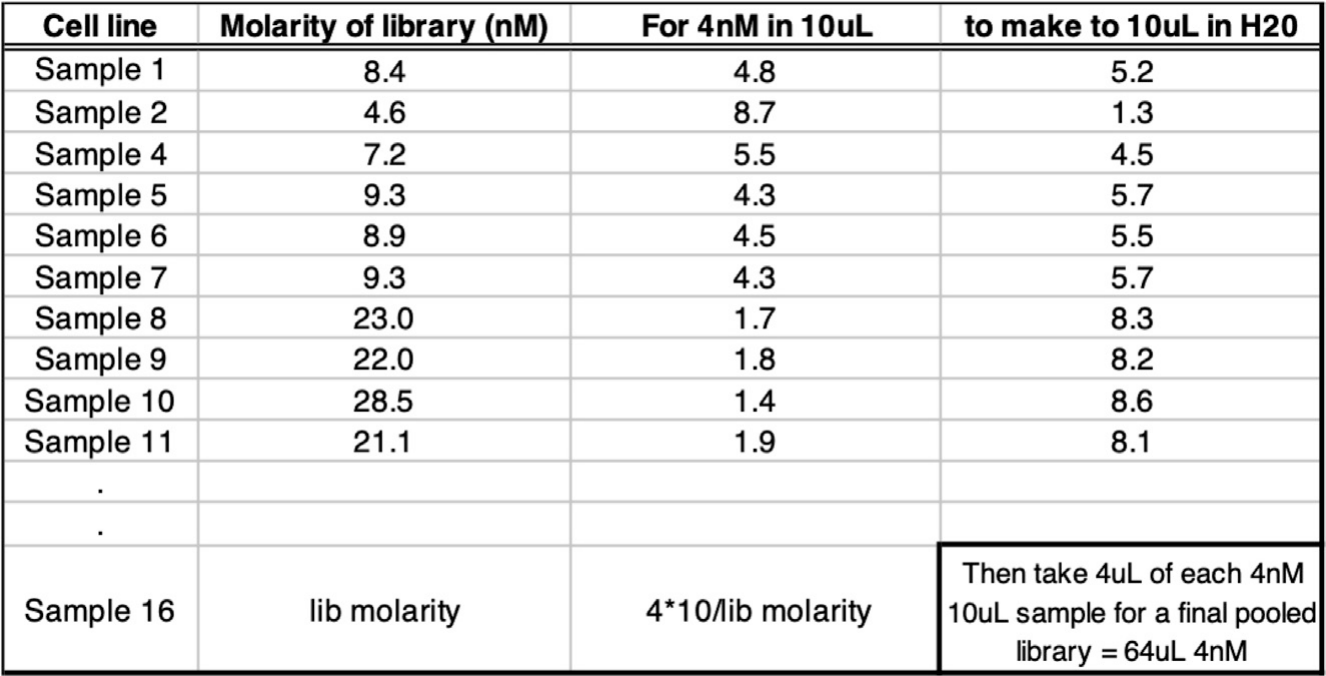
Example of How to Pool the ATAC-Seq Library for Sequencing

52. Run HS DNA chip on Bioanalyzer and quantify library molarity using the bioanalyzer software: 2100 Expert Software (Figures 2 and 3).

***Note:*** Download for free from: https://www.agilent.com/en/product/automated-electrophoresis/bioanalyzer-systems/bioanalyzer-software/2100-expert-software-228259

**Pause Point:** Libraries can be stored individually at −20°C.

**CRITICAL:** To prevent index hopping, libraries should be pooled just before sequencing. For further details please refer to https://emea.illumina.com/content/dam/illumina-marketing/documents/products/whitepapers/index-hopping-white-paper-770-2017-004.pdf.

## Expected Outcomes

A successful ATAC-seq library will typically have a molarity in the region of 4–30 nM (Figure 3).

Successful libraries typically require fewer than 12 total cycles of PCR amplification. More than 12 cycles can impact the library complexity and result in over-amplification of the same DNA fragments. Library complexity can be improved by making biological replicates and selecting the best libraries, i.e., libraries that require the minimum number of amplification cycles (Figure 1) or by optimizing the cell input.

**Figure 4:**
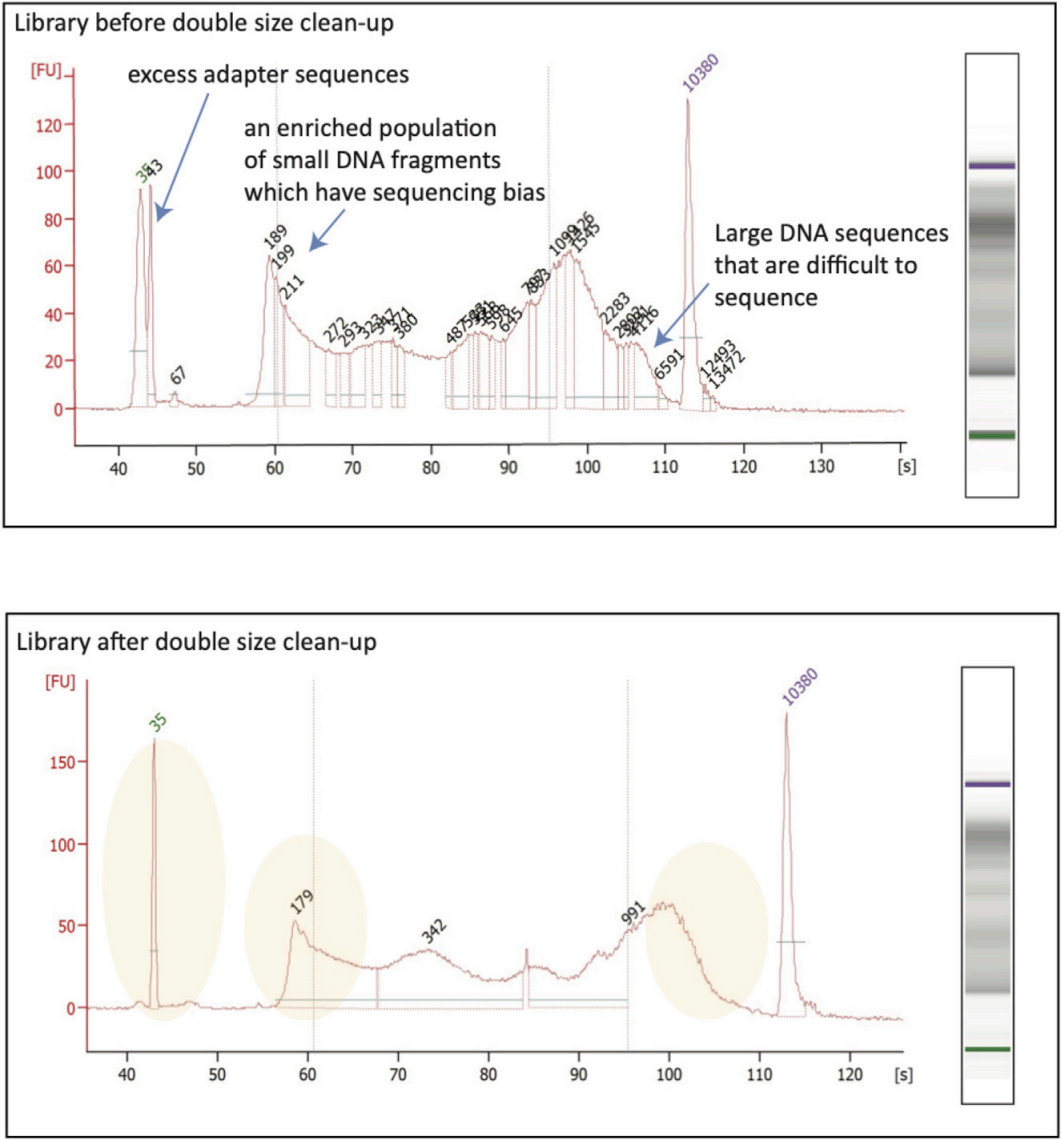
Bioanalyzer Result of an ATAC-Seq Library before and after Double Size Selection Note that excess adapter sequences have been removed. Large DNA fragments (>1,000 bp) and small DNA fragments (<100 bp) are reduced.

Successful double size selection results will contain a selection window ranging from 100–1,000 bp (Figure 4). The full fragment size distribution should be maintained.

## Quantification and Statistical Analysis

### Library Sequencing

**Timing: 48 h**

**Figure 5:**
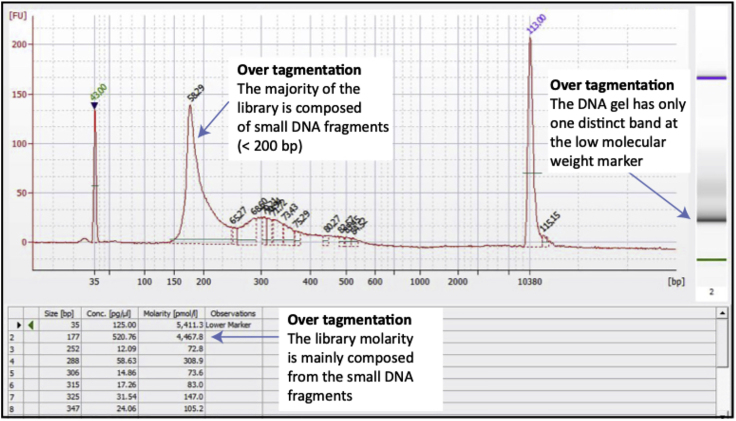
Bioanalyzer Result Demonstrating Over-Tagmentation

ATAC-seq and the Nextera workflows are designed for sequencing using Illumina high-throughput sequencing instruments. When sequencing ATAC-seq libraries, Nextera-based sequencing primers and reagents must be used Figure 5). For inferring differences in open chromatin with human samples and transcription factor mapping we used >100 million mapped reads.

1. A 4 nM library pool of 16–20 libraries were clustered and sequenced over 8 lanes of an Illumina HiSeq 4000 flow cell. 5 μL of the 4 nM library pool was used for each lane. Clustering was done onto the flow cell by using a HiSeq 3000/4000 cluster kit and an Illumina cBot2.

**CRITICAL:** To achieve the best quality of sequence avoid generating air bubbles when clustering reagents are mixed.

2. 75 bp paired-end sequencing was performed on an Illumina HiSeq 4000 sequencer.

***Note:*** If a different sequencing platform will be used for sequencing of the library pool the molarity of the pool and the number of libraries in the pool may have to be adjusted.

***Note:*** Data yield can be impacted by the large fraction of mitochondrial reads.

### Sequence Read Mapping and Peak Calling

**Timing: 4–8 h**

Stand-alone bioinformatic analysis pipelines for ATAC-seq read mapping, quality control, peak calling, and differential analysis include:

(i) the *bcbio-nextgen* toolkit (via the *ChIP/ATAC-seq* pipeline) https://bcbio-nextgen.readthedocs.io/en/latest/contents/atac.html; or
(ii) the nfcore-atacseq pipeline https://nf-co.re/atacseq.

These pipelines are capable of running tasks across multiple compute infrastructures. Importantly, both pipelines incorporate QC analysis steps to determine whether your ATAC-seq experiment is suitable for further downstream analysis. In addition to alignment level QC and estimation of library complexity, ATAC-seq specific QC reports are generated using *ataqv* https://github.com/ParkerLab/ataqv. ataqv produces a web-based visualization and analysis report including a number of basic QC metrics.

The authors recommend that practitioners follow ATAC-seq standards used by ENCODE https://www.encodeproject.org/atac-seq/ and refer to the following guide which provides a detailed overview of the ATAC-seq analysis workflow and important QC metrics https://yiweiniu.github.io/blog/2019/03/ATAC-seq-data-analysis-from-FASTQ-to-peaks.

As a general rule, experiments should have two or more biological replicates with each replicate having 50 million non-duplicate, non-mitochondrial aligned reads when using paired-ended sequencing. Mitochondrial reads will typically have high coverage and should be removed. Duplicate reads which likely arise from PCR artifacts should also be removed. The percentage of mapped reads should be greater than 80%.

ATAC-seq specific quality metrices can also be evaluated using ATACseqQC: a Bioconductor package for post-alignment quality assessment of ATAC-seq data Ou et al., 2018. Successful mapping of ATAC-seq libraries will be in the region of 60%–90%.

https://bioconductor.org/packages/release/bioc/html/ATACseqQC.html

3. Set up a YAML file detailing the samples and the pipeline to use

***Note:*** This pipeline only requires a YAML file. Installation of the *bcbio-nextgen* toolkit, setup of the YAML file and running of the pipeline is well documented in the *bcbio-nextgen* manual (https://bcbio-nextgen.readthedocs.io/en/latest/index.html).

4. Remove any samples with a low number of mapped reads (we used a threshold of <100 million mapped reads from the pipeline QC report) to exclude samples from the downstream analysis
5. Check output peak files from *macs2* component of *bcbio-nextgen* pipeline for successful peak calling

***Note:*** The number of peaks called will vary by sample but we would expect a minimum of 10,000 peaks called for most samples.

### Differential Peak Analysis and TF Mapping

Peaks associated with specific conditions are identified by differential peak analysis. Enrichment of condition-specific transcription factors is calculated by identifying transcription factor binding motifs in the center of condition-specific peaks.

***Note:*** Bioconductor package *DiffBind* (https://bioconductor.org/packages/release/bioc/html/DiffBind.html) is used for the condition-specific peak identification and *HOMER* (http://homer.ucsd.edu/homer/motif/) is used for the TF mapping. Manuals are available for both software detailing all options available for each step.

6. Create a sample sheet detailing all samples. This can be an *R* data frame or a CSV/XLSX file and must include sample ID, condition, replicate (if applicable), location of BAM file and location of *macs2* peak file (see *DiffBind* vignette and user manual for further options).
7. Read in the peaksets for all samples using the *dba()* function. The only input required for this function is the sample sheet from step 6 (either (a) a data frame containing the sample sheet or (b) the name and location of CSV/XLSX file to load).
library(DiffBind)
a) dba.object <- dba(sampleSheet = sample.sheet)
b) dba.object <- dba(sampleSheet = “path/to/sample_sheet.csv”)
8. Count reads for the full set of peaks called across all samples (*dba.count()*). Re-center peaks around the most enriched point of the peak and set to a standard width of 500 bp (250 bp up- and 250 bp downstream of peak center) using the option “*summits = 250*”.
dba.object <- dba.count(dba.object, summits = 250, score = DBA_SCORE_TMM_READS_FULL)

***Note:*** Various exploratory plots can be generated to visually inspect the data. For example, correlation heatmaps can be generated after both steps 7 and 8 to produce a clustering of the samples (*plot(dba.object)*) and a PCA plot can be generated after step 8 (*dba.plotPCA(dba.object, DBA_CONDITION, label = DBA_ID)*).

9. Set up the contrasts for the comparisons of interest (*dba.contrast()*). The groups required for the contrast should be specified in the “*condition*” field of the sample sheet.
dba.object <- dba.contrast(dba.object, categories = DBA_CONDITION, minMembers = 2)
10. Perform differential peak analysis with *dba.analyze()*
dba.object <- dba.analyze(dba.object, method = DBA_DESEQ2)
11. Extract condition-specific peaks with *dba.report().* Peaks differentially open in one condition compared to another condition(s) are reported (FDR <= 0.05). Peaks can be classified as open in one condition compared to another based on the values in the Fold column and the condition concentration columns.
dba.report.object <- as.data.frame(dba.report(dba.object), contrast = 1)
peaks.condition1 <- subset(dba.report.object, Fold > 0)
peaks.condition2 <- subset(dba.report.object, Fold < 0)

***Note:*** If there are more than 2 conditions being compared “*contrast =*” should be changed accordingly. Peaks in *peaks.condition1* are “open” in condition 1 (“closed” in condition 2) while peaks in *peaks.condition2* are “open” in condition 2 (“closed” in condition 1).

12. Produce BED files (or *HOMER* peak files) of the genomic locations for the open peaks in each condition (see *HOMER* manual for details of input file format: http://homer.ucsd.edu/homer/ngs/peakMotifs.html).
13. Perform TF motif enrichment for each condition using the *findMotifsGenome.pl* script from the *HOMER* software. The only required user input is the peak file. Other required arguments of the function are:
genome (e.g., hg19)
output directory
size
(optional: user background peak file - BED file or HOMER peak file. *-bg background/BED file*)
findMotifsGenome.pl <peak/BED file> <genome> <output directory> -size # [options]

***Note:*** “*size*” was set to 200 to focus motif detection on the 200 bp around the center of each peak.

***Note:****findMotifsGenome.pl* can compare identified motifs in the input BED file to a randomly produced background or a user supplied background file. We found that comparison to a random background consistently reported AP-1/JUN factors as the most enriched motifs. Comparison of open peaks to closed peaks in one condition produced condition-specific results.

### Promoter Mapping and GO Enrichment Analysis

Downstream functional analysis of normalized ATAC-seq data was performed using Bioconductor packages *ChIPseeker*, and *clusterProfiler*.

14. To map chromatin accessibility peaks to genomic annotations the *annotatePeak* function from the ChIPseeker package was used. This function uses as input the diffBind dba.report.object and annotation packages *TxDb.Hsapiens.UCSC.hg19.knownGene* and *org.Hs.eg.db* to map peaks to known genes. The transcriptional start site is also defined by the *tssRegion* argument, which in the example below, encompasses 3,000 bp upstream and downstream of the transcriptional start site.
library(ChIPseeker)
library(TxDb.Hsapiens.UCSC.hg19.knownGene)
library(org.Hs.eg.db)
txdb <- TxDb.Hsapiens.UCSC.hg19.knownGene
annotated,PeakList <- annotatePeak(dba.report.object, tssRegion=c(-3000, 3000), TxDb=txdb, annoDb="org.Hs.eg.db")

The successful execution of this function generates an annotated peak list object which can be subset to obtain peak sets specifically located within promoter, intronic or distal intergenic regions. For example,

distalPeaks <- subset(annotatedPeakList@anno, annotation=="Distal Intergenic")
15. The unique set of genes associated with these specific peak sets can then be used as input for gene set enrichment analysis. The Bioconductor/R package *clusterProfiler* can be used to identify genes associated with specific peak sets that are enriched in KEGG and/or Reactome pathways.
library(ReactomePA)
library(clusterProfiler)
library(org.Hs.eg.db)
keggResults <- enrichKEGG(gene = as.data.frame(distalPeaks)$geneId, organism = 'hsa')
dotplot(keggResults, showCategory = 30)
reactomeResults <- enrichPathway(gene = as.data.frame(distalPeaks)$geneId)
dotplot(reactomeResults, showCategory = 30)

## Limitations

Both genomic DNA and mitochondrial DNA are processed in ATCA-seq libraries. To ensure enough sequencing depth to allow for TF mapping and removal of contaminating mitochondrial reads, faster sequencing platforms such as NextSeq 500 mid output flow cells (130 million) reads provided insufficient sequencing depth and problems with over-clustering. Therefore, sequencing platforms such as NovaSeq 6000 or NextSeq 2000 are recommended. The Illumina HiSeq system is also suitable for sequencing ATAC-seq libraries but has recently been discontinued.

## Troubleshooting

### Problem

Over- or under-tagmentation (visible by bioanlayzer electropherogram).

### Potential Solution

**Figure 6:**
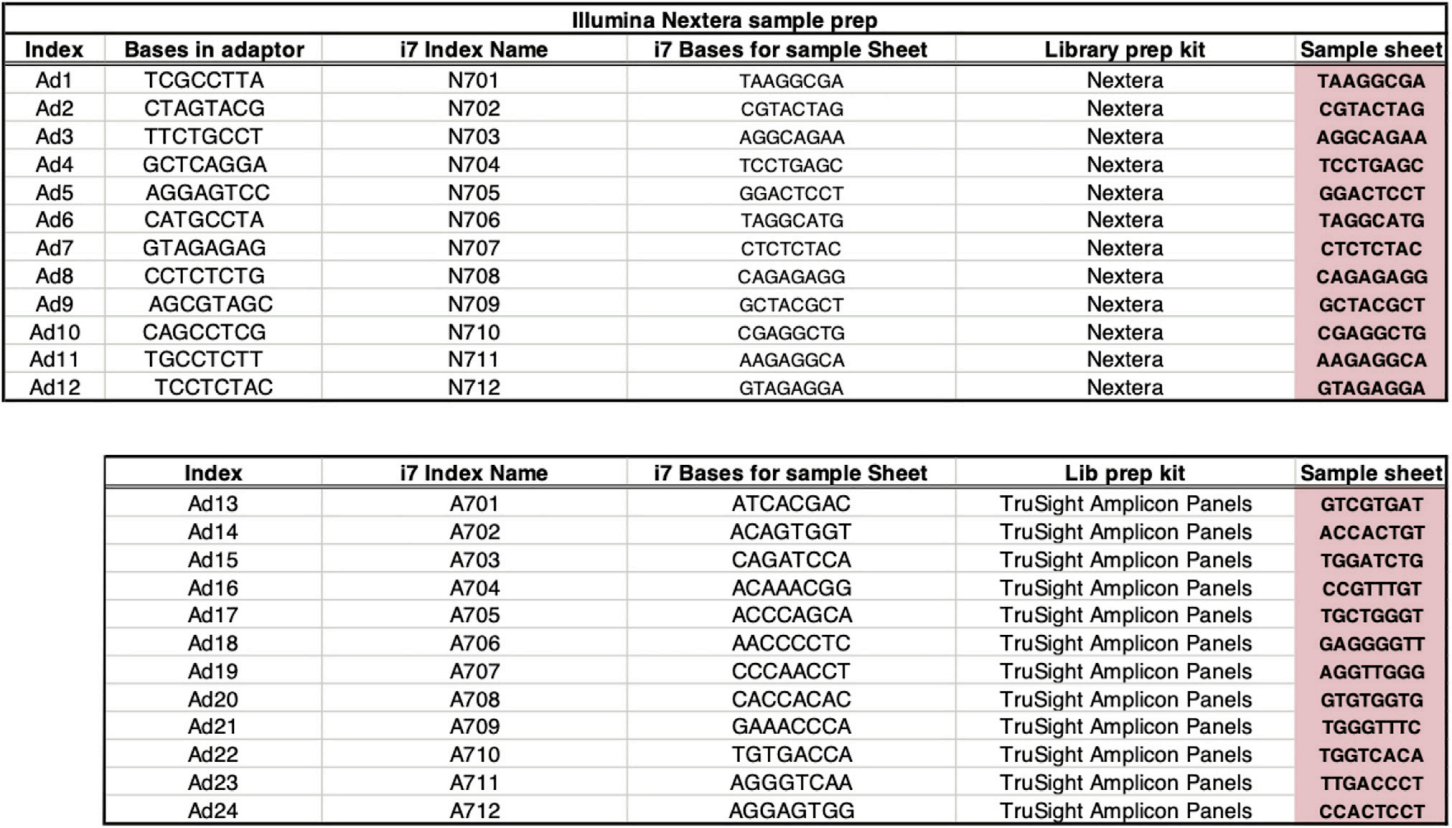
Bases in Adapter Sequence that Identify the Unique Sample Index Highlighted in pink are the unique identifying sequences for sample identification.

Most problems arise from not determining the appropriate number of cells to use in the tagmentation reaction. A low cell to transposase enzyme ratio can result in over-tagmentation, which produces a library mainly represented by small DNA fragments (Figure 6).

Over-tagmented libraries can be identified by a DNA trace that has one distinct band of sub-mononuleosomal size and has lost banding patterns at higher molecular weight bands representing mono-nucleosomal and di-nucleosomal sizes.

Under-tagmentation occur when too many cells have been added and results in a library mainly composed of large DNA fragments, which can be identified by one distinct band at the higher molecular weight marker on a DNA gel.

To resolve the problem of under- or over-tagmentation first try a series of cell inputs for the transposition reaction. Typically, a range between 50,000–200,000 cells worked well for PDCLs tested. Different cell types may require less or more cells for this reaction.

### Problem

Persisting small or large DNA fragments after the two-part size selection library clean-up (visible by bioanlayzer electropherogram).

### Potential Solution

Unwanted DNA fragments can persist after the SPRIselect clean-up step which can be a consequence of either bead contamination or incorrect incubation time with the beads.

For large DNA fragment contamination, be careful not to pipette any of the beads that are attached to the magnet when removing the library supernatant. If beads detach into the library supernatant, place the Eppendorf tube back on the magnetic rack and wait for all beads to reattach to the magnet. Then carefully remove the library supernatant.

If bead contamination is not the problem, extending the time of DNA and bead incubation can increase binding of DNA fragments. However, this should be optimized to ensure that DNA fragments of interest do not also bind. Typically, an incubation time in the region of 5–15 min is recommended.

For small DNA fragment contamination, the ratio of SPRIselect can be optimized to exclude smaller DNA fragments. For example, a ratio between 1.8 – 1.2 SPRIselect will bind fragments in the region of 100–1,000 bp, however this may vary depending on cell type.

### Problem

DNA smear (visible by bioanlayzer electropherogram)

### Potential Solution

A DNA smear can be caused by violent pipetting during the lysis step. During the cell preparation and transposition steps (step 4–13) treat the cell pellet very gently. Lightly dislodge the pellet by flicking the Eppendorf tube rather than by pipetting. If pipetting is required, carefully resuspend the pellet once. Do not aggressively resuspend the pellet in lysis buffer by pipetting multiple times.

Additionally, if the level of detergent is too high for your specific cell type the nucleus might rupture, therefore the detergent concentration might require optimization for different cell types.

### Problem

Low DNA yield.

### Potential Solution

Significant library yield can be lost during the two-part size selection library clean-up. Library incubation times with beads must be optimized to ensure the correct sizes are captured (as described above).

During the SPRIselect bead ethanol wash step (step 33), it is crucial not to remove beads during this step because the desired library is now associated with the beads.

Increasing the cell input should also increase the DNA yield and should be optimized for different cell types.

## Resource Availability

### Lead Contact

Further information and requests for resources and reagents should be directed to and will be fulfilled by the Lead Contact, Dr Peter J. Bailey (Peter.Bailey.2@glasgow.ac.uk).

### Materials Availability

The patient-derived cell lines were provided by the Australian Pancreatic Cancer Genome Initiative (APGI, www.pancreaticcancer.net.au) and the Garvin Institute of Medical Research (Sydney, Australia).

### Data and Code Availability

ATAC-seq sequencing data from patient-derived cell lines can be found at BioProject ID: PRJNA630992. Original data for all datasets in this paper are available at Mendeley Data DOI:10.17632/74s7crj7xj.1. All software packages used are publicly available through commercial vendors.
